# Supplementary material for: How do question-answer exchanges among generations matter for children’s happiness?
Source: PLoS One. 2024 Jun 21;19(6):e0303523. doi: 10.1371/journal.pone.0303523 (PMC11192389; doi:10.1371/journal.pone.0303523)

Our responses to reviewer 2 regarding the manuscript "How do question-answer exchanges among generations matter for children's happiness?" submitted to *PLOS ONE*

First, thank you for spending the time on the manuscript and providing us with your comments. Your comments are very constructive and useful for the improvement of our paper. We have tried to clarify all of your quarries and modified our manuscript, accordingly. Our responses to your comments are given below.

Reviewer 2's general comments and my responses

1. **Reviewer comment**: The research questions addressed in this manuscript are both novel and important. However, the rationale for the questions in the Introduction is sometimes difficult to follow; for example, there are some distracting claims in the introduction (e.g., the statement about Maslow, noted below), and key terms (e.g., "generative concern") need to be explained more clearly and directly early on in the Introduction. A general (high-level) orientation to the methodological and analysis approach for the study in the final part of the Introduction would also be valuable. Elaboration on these and additional general points, and more specific comments, are provided below.

   Our response: We greatly appreciate your courteous and constructive comments. We have learned a lot from your comments. We have revised the manuscript as much as possible in response to your specific ideas and suggestions. We have responded to each of your suggestions as follows.

2. **Reviewer comment**: Introduction: The meaning of the phrase "generative concern" is not clear early on in the Introduction. Please explicitly state what is meant by this phrase early in the Introduction (e.g., in the first paragraph). For example, on page 6 it is characterized as "offering care for people and (the) natural environment." Providing a few examples/paraphrases of the actual questions given to the young age-group tested could also be helpful.

   Our response: Thank you for your kind comments. We understand and agree with your points. Accordingly, We have added the meaning indicated by "generativity" to the first paragraph of the Introduction and revised it as follows.

   [***Introduction section: Page 1, lines 2-16***: *Curiosity is an essential element of individuals' creativity, maturity and open-mindedness [33, 55, 32, 22]. Children's tendency to ask questions, driven by their curiosity, shall be an initial step in building human relations and learning. In the literature, such a tendency is conceptualized as "inquisitiveness" representing curiosity about something and someone different [35, 19]. For instance, [18] examine adult-child conversational exchanges by focusing on young children's questions and responses and claims that such communications provide essential bases for children's future life, especially their growth through human interactions. Regarding the role of adults in responding to children's curiosity, "generativity" has been known as the characteristic that helps the next generation grow and lead to a sustainable society [17, 45, 56, 26]. Moreover, having excellent relationships with family, friends and others contributes to people's generativity and happiness [45, 28, 27, 53, 60]. However, little is known about how inquisitiveness influences*

people's generativity and happiness in their childhood. Therefore, this research addresses the role and mechanism of children's inquisitiveness in the emergence of generativity and their happiness through intergenerational communication.] ■

3. **Reviewer comment**: Introduction: It is a considerable mischaracterization of Maslow's theory to suggest that "people are happy as they become wealthy." Please rephrase.

   Our response: Thank you for your precise comments. We agree with your points. Our description is written in a way that misleads the reader. Accordingly, We have revised the relevant text as follows.

   [***Introduction section: Pages 1-2, lines 17-32***: *Wellbeing represents the outcome of a "good life," where people are assumed to act and behave as happiness seekers [47, 30, 40, 20]. Maslow [44] is the psychologist who proposed a wellbeing theory, i.e., Maslow's hypothesis, based on psychological needs and gratification processes, and implies that material wealth increases people's satisfaction with the basic needs stage but that other factors, such as psychological factors, are essential to people's overall wellbeing. To examine this hypothesis, some researchers have developed and refined happiness measurements, such as the subjective happiness scale (SHS) and satisfaction with life scale (SWLS) [see, e.g., 9, 10, 41]. [59] and [8] empirically examine the hypothesis with cross-country data utilizing happiness scales and conclude that wealth can explain variations in happiness across countries; however, there should be some other crucial predictors. Following these works, the literature has focused on how happiness is associated with various cultural, sociodemographic and personal factors other than income or wealth, including gender, marital status, educational record, self-esteem, optimism and human relations [12, 13, 31, 36, 29, 49, 11, 4]. Overall, aging, income, human relationships and personality traits are established to be the essential characteristics of happiness [62, 65, 39, 2, 46, 42, 1].*] ■

4. **Reviewer comment**: The subsection on "Participants" is an admixture of study overview statements, participants, and procedure.

   (a) It should be greatly condensed, using more direct and simple statements, that specifically describe the participants.

   (b) It could be helpful for some of the demographic information that is presented in the first section of the results (e.g., percentage of females and mean age in urban and non-urban areas) to be instead provided here in the Participants subsection.

   (c) Please indicate how many participants were invited to take part vs. how many actually participated.

   (d) Some of the text in the Participants subsection could be better presented earlier, as a study overview or similar (e.g., the broad characterization of the measures and the reference to Figure 1). Perhaps provide such an overview in a paragraph or two before the Method section.

   (e) Some of the text in the Participants subsection, such as sending invitation letters and obtaining written consent should be in a separate Procedure subsection.

   (f) The procedure subsection should also provide additional details of how the study was actually conducted, such as how long it took for the children to complete the questionnaires,

the order in which the questionnaires were completed, when the questionnaires were administered (e.g., in the children's classrooms, during class-time or before or after school) and how they were administered (e.g., in computerized or paper-and-pencil format).

Our response: Thank you for your kind and thoughtful comments. In accordance with your suggestions (a) to (f), We have modified the manuscript as follows: (a) We have tried to modify the participant subsection to be greatly condensed that specifically describe the participants; (b) We have modified the "Participants section" to incorporate some of the demographic information from the Results section into the description; (c) We have revised the text to add that a total of 570 invitations were sent to the three study sites, with a final total of 511 participants; (d) We have added a description in the last paragraph of the introduction that serves as an overview of the study. We modified the text of the relevant paragraphs by adding some of the data measurement descriptions presented in the Participants subsection; (e) We have described the survey invitation and consent form in the newly added Procedure subsection; (f) We have added additional information to the Procedures subsection, including where the survey was administered, when it was administered, the order of the questions, the time required to answer, and how it was administered.

[***Participants subsection: Pages 4, lines 102-108***: *The participants in the study are elementary and junior high school students living in a certain municipality in Kochi Prefecture, Shikoku, Japan. The participants' mean age is* 12.32 *years with the standard deviation* $=$ 14.23, *ranging between* 9 *and* 14 *years. The percentage of female participants and their mean age are almost similar in urban and non-urban areas,* 46 % *and* 47 %, *as well as* 12.61 *years and* 12.21 *years, respectively. We have surveyed three regions (one categorized as urban and two non-urban) for the study because they possess different sociodemographic and geographical characteristics.*] ■

[***Procedure subsection: Page 4, lines 110-126***: *We recruited the participants by sending them invitation letters from the municipal education board offices. A total of 570 invitations were sent to schools in the three study sites. We collected* 511 *samples in city-A (*140*) and two rural areas of town-B (*225*) and town-C (*146*), respectively. After obtaining written consent for participation from the individual and his/her parents, we conducted a questionnaire survey in several school facilities in the study regions. The questionnaire used simple language that elementary school students could understand and was designed to be easy to answer on a touchscreen display. This survey collects a sample of* 511 *participants with information by asking them in the following order: (*1*) subjective happiness; (*2*) inquisitiveness (curiosity about and acceptance of something and someone different and/or new); (3) the revised generative concern (offering care for people and the natural environment); (4) costs/benefits of the consultation (positive effects of reaction in an appropriate manner) and (5) sociodemographic factors, such as age, gender, attending tutoring school, joining sports clubs and household members. The variables we collected in this survey can be categorized into cognitive, non-cognitive and sociodemographic factors in relation to subjective happiness, as described in Figure 1. The data were collected between November and December 2021.*] ■

[***Introduction section: Pages 2-3, lines 62-83***: *No previous works have addressed how children's happiness is characterized by inquisitiveness and generativity, while these con-*

cepts are known to be concerned with how people build and keep good relationships with their family, friends and others. Inquisitiveness is considered an essential factor that triggers communication even in a child and contributes to building pleasant human relations. Therefore, even among children, inquisitiveness is hypothesized to be a crucial determinant of happiness and generativity. This study aims to empirically examine the cognitive, noncognitive and sociodemographic factors of inquisitiveness, generativity and happiness in children using a single analytical framework, as described in Figure 1. Thus, we conduct questionnaire surveys with 511 *Japanese participants between 9 and 14 years of age to collect data, following previous studies that have analyzed the relationships between behaviors and happiness with cross-sectional data [58, 61, 37, 51, 52]. The study collects data to analyze the relationship between children's curiosity, adult responses to children's questions, children's attitudes toward the younger and nature and children's happiness using the following measures: inquisitiveness (curiosity about and acceptance of something and someone different and/or new); costs/benefits of the consultation (positive effects of reaction in an appropriate manner); the revised generative concern (offering care for people and the natural environment); and subjective happiness. With these data, this study addresses the following three research questions. (1) How does children's inquisitiveness, along with generativity, affect their happiness? (2) Does their inquisitiveness play a role in generativity? (3) How does adults' manner of responding affect children's inquisitiveness?*] ■

5. **Reviewer comment**: Discussion – The first paragraph of the discussion, which has multiple specific references to Figure 2, seems more appropriately placed as a concluding paragraph of the Results.

   Our response: Thank you for your thoughtful comments. I understand and agree with your points. Accordingly, I have moved the relevant paragraph in the "Discussions" section to the last paragraph of the "Results" section and modified it as follows.

   [***Results section: Pages 17-18, lines 489-505****: We now summarize the answers to the three research questions at the end of the introduction. As described in our conceptual framework of Figure 1, it is well known that cognitive, noncognitive and sociodemographic factors mainly characterize happiness. The first question is, "How does children's inquisitiveness, along with generativity, affect happiness?" We answer that generativity* (α_1)*, inquisitiveness* (α_2) *and adult's positive response* (α_3)*, directly and indirectly, affect happiness in childhood, demonstrating the importance of generativity and inquisitiveness for subjective happiness (SH) in Figure 1. The second question is, "Does children's inquisitiveness play a role in generativity?" We answer that inquisitiveness* (β_1) *is meaningful to ascertain whether children possess the signs of generativity, along with adult's positive response* (β_2) *in Figure 1. The third question is, "How does adults' response manner affect children's inquisitiveness?" We answer that adult's positive response* (γ_1) *is the crucial determinant to ascertain whether children possess high inquisitiveness in Figure 1. Previous studies indicate the importance of generativity for an adult's subjective happiness [54, 25, 26]. However, our regression and SEM analyses show that the magnitude and statistical significance of inquisitiveness and adults' positive responses are more important for subjective happiness in their childhood than generativity.*] ■

6. **Reviewer comment**: Discussion – The sentence, "Our study demonstrates that children

who receive suitable responses from adults tend to increase their inquisitiveness and express the signs of generativity" is an over-statement of what the study can show (given it is an entirely cross-sectional study, using only self-reported outcomes). What the study shows is that (at one point in time) children's self-reported inquisitiveness is strongly positively associated with their (self-reported) perception of receiving encouraging responses for their inquisitiveness from adults, and also is positively associated with children's (self-reported) generativity concerns.

Our response: Thank you for your kind comments. As you indicated, our study is cross-sectional. The data collected are self-reported by questionnaire at a certain point in time, and the relevant sentence is an over-statement. Accordingly, we have revised it as follows.

[***Pages 18-19, lines 524-546**: Hirose and Kotani [25] and Hirose et al. [26] have pointed out that inquisitiveness positively correlates with how people enhance their generative concern and happiness through questionnaire surveys with Japanese and Palauan adults, respectively. According to Hirose and Kotani [25] and Hirose et al. [26], inquisitiveness, directly and indirectly, affects happiness, but the most critical factor is generativity. Our study also demonstrates that children's degree of inquisitiveness is positively correlated with their generativity and happiness. Some studies have pointed out that inquisitiveness is stable as a part of one's critical thinking disposition [5, 7, 3]. Conversely, other studies have pointed out that inquisitiveness can be acquired and further enhanced by learning [24, 18, 64, 19].*

Inquisitiveness and adults' positive responses are identified as the main determinants of children's subjective happiness, and our regression models show generativity as insignificant. Generativity is the characteristic that helps and leads the next generation to grow [17, 45, 56, 26]. Childhood is also important as a preparatory process for generativity, but for children, it is more critical to arouse and satisfy inquisitiveness through good relationships with adults than with the problems of future generations. Although limited by data from self-reported answers at one point in time, our study demonstrates that children's inquisitiveness is strongly and positively associated with the perception that they received an encouraging response to their inquisitiveness from an adult and with signs of their generative concern. Overall, the results of this study suggest that learning together in intergenerational relationships between adults and children is essential for people's subjective happiness and should be considered for development as an educational program for families, communities and schools.] ■

7. **Reviewer comment**: What is to be made of the negative relation between children's inquisitiveness and children's age (e.g., page 17 and Table 5)? There should be some explicit discussion of this outcome.

   Our response: Thank you for your constructive comments. We agree with the points you raised. The negative correlation between age and inquisitiveness is consistent with some previous studies [5, 57, 15, 6]. Other studies have reported on extrinsic curiosity enhanced by prompting by educators [38, 50]. While our study's data set is insufficient to explain the negative correlation between age and inquisitiveness, we have provided one of statistical evidence that adult responses to children's questions may be related to their curiosity. Accordingly, we have added a sentence in the Result section and one paragraph in the Discussion section to explain the negative relationship between age and inquisitiveness as follows.

[***Results section: Page 15, lines 430-447***: *Second, age negatively affects inquisitiveness at* 1 % *significance in models 1 and 4. The estimated coefficients of age in models 1 to 4 suggest that the participants may have a decrease in the range of their inquisitiveness between* -0.770 *and* -0.957 *when they age by one year. The negative value indicated by age is consistent with several studies that report a negative linear transition of curiosity, a concept similar to inquisitiveness, once young children begin formal schooling [57, 15, 6]. "Family food shopping" also exhibits* 5 % *statistical significance with a positive sign in models 2 to 4, implying that the participants who go grocery shopping with their parents enhance their inquisitiveness* $0.324 \sim 0.383$, *as compared with other participants. Several independent variables, such as "joining sports clubs," "negative response," gender and areas show the possibility as minor determinants of the participants' inquisitiveness. The other independent variables, such as household members and tutoring school days, are statistically insignificant, as shown in models 2 to 4 in Table 6. We confirm that the main results qualitatively remain the same, irrespective of the various specifications of models other than models 1 to 4, such as interaction terms among the variables. Overall, adult's positive response, age and "family food shopping" are confirmed as the main determinants having statistical and practical significance to the likelihood of the participants increasing their inquisitiveness.*] ■

[***Discussions section: Page 18, lines 507-523***: *We should mention the inverse correlation between the children's age and their inquisitiveness, shown in Table 6. The negative correlation, as revealed by the regression analysis in our study, is consistent with several previous studies reporting a linear negative transition between children's age and curiosity [57, 15, 6]. Many studies report a general decline in questioning throuthout children and adolescence [16, 21, 5, 57, 15]. For instance, Tizard and Hughes [57] find that children reduce their questions from twenty-six an hour at home with their mothers to two per hour upon entering the formal education system. While we understand the importance of distinguishing curiosity from inquisitiveness, both share concepts that help us predict the nexus between age and inquisitiveness. Other studies cast doubt upon the veracity of the purported linear transition in age and curiosity, advocating instead for the pivotal role of educators and environmental factors in nurturing children's inquisitive disposition [38, 50]. Regrettably, our dataset remains insufficient to reveal the mechanism of the negative correlation between children's age and their inquisitiveness. Nevertheless, our study proffers empirical evidence that suggests the hypothesis that constructive adult responses to children's inquiries may positively correlate with the young ones' inquisitiveness.*] ■

8. **Reviewer comment**: Minor Comments

(1) – p. 2, a manuscript submitted to a scientific journal would not usually include a"content" page (a contents page is generally more expected/appropriate for a book or a thesis)

(2) – p. 5, words missing/fragmented sentence: "Therefore, inquisitiveness is hypothesized to be a crucial determinant of happiness and generativity, even among children, and empirically examine their cognitive, noncognitive and sociodemographic factors using a single analytical framework." Suggest separating these key ideas into two sentences, along the lines: "Therefore, inquisitiveness is hypothesized to be a crucial determinant of happiness and generativity, even among children. The aim of this study is to empirically examine the cognitive, noncognitive and sociodemographic factors of inquisitiveness, generativity and

Table 6: Estimation results of median regression on child's inquisitiveness

Variable	Inquisitiveness			
	Model 1	Model 2	Model 3	Model 4
Adult's positive response	0.717***	0.673***	0.680***	0.657***
	(0.046)	(0.056)	(0.057)	(0.056)
Age	−0.770***	−0.901***	−0.904***	−0.957***
	(0.210)	(0.242)	(0.246)	(0.244)
Family food shopping		0.383**	0.324**	0.377**
		(0.150)	(0.151)	(0.146)
Joining sports clubs		0.914	1.099	1.536**
		(0.686)	(0.718)	(0.709)
Adult's negative response		−0.074	−0.059	−0.041
		(0.073)	(0.074)	(0.073)
Gender (base group = male)			0.885	1.273*
			(0.712)	(0.702)
Household members			−0.115	−0.323
			(0.269)	(0.265)
Tutoring school days				0.135
				(0.210)
Area (base group = non-urban)				−1.251
				(0.783)

***significant at 1 %, **significant at 5 percent,*significant at 10 percent

happiness in children (9 to 14 years of age) using a single analytical framework."

(3) – p. 7, the subsection heading should read "Measures"

(4) – p. 9 - p. 10, the word "theoretical" in "theoretical range" (several places) is confusing; suggest rephrasing as "the maximum possible range"

(5) – p. 10: "These subscales are reliable for influencing people's behaviors and attitudes in many vital contexts" – suggest rephrasing to read something like, "These subscales have been found to reliably assess people's behaviors and attitudes in many important contexts"

(6) – p. 11 - p. 12 – rephrase "disturbance term" (several places) as "error term"

(7) – p. 12 – the rationale for performing median regression analyses might be better placed earlier in the results section.

(8) – p. 15 - p. 16: important typo: the text here should read "Overall.... confirmed to be the main determinants of the participants' subjective happiness (SH)" ("not of the participants' generativity")

(9) – p. 18 – the relevant Figure number for path C is not indicated

(10) – p. 18 – the subsection heading should read "Discussion"

(11) – p. 18 – suggest rephrasing to read "the three research questions posed"

(12) – p. 19 – the text, "children's inquisitiveness statistically affects the sign of generativity and happiness" is unclear/difficult to parse; suggest something like "children's degree of inquisitiveness is significantly positively correlated with their generativity and happiness"

(13) – p. 20 – the reference to "panel data" is unclear; suggest "longitudinal data" or similar

(14) – Figure 2 – the text in the Figure should read "sports clubs" (not ports clubs)

Our response:

Thank you for your valuable comments. Your comments are very valid, and we agree with all of them. Accordingly, we have addressed and modified each comment as follows.

(1) Thank you very much for your appreciative comments. We have gladly learned from your kind comments. We will now put what we have learned to good use.

(2) Thank you for your kind comments. We agree with splitting the sentence into two parts to make it easier to understand. Accordingly, we have revised the relevant sentence as you suggested.

(3) Thank you for your comments. I agree with your comments. Accordingly, I have corrected the subsection"Measur" to "Measure" in the manuscript.

(4) Thank you for your comments. We are glad to hear your honest opinion that using"theoretical" is confusing. We have followed your suggestion and rephrased the"theoretical range" to"the maximum possible range."

(5) Thank you for your kind comments. We completely agree with your remarks. The scale has come to be trusted as a measure of people's behavior and attitudes. Accordingly, we have revised the text as follows."These subscales have been found to reliably assess people's behaviors and attitudes in many important contexts."

(6) Thank you for your comments. We are glad to hear your opinion that the“disturbance term” is not appropriate to use. We have followed your suggestion and rephrased the“disturbance term” to“error term.”

(7) Thank you for your comment. We have thought it over and agree with you. Accordingly, we have moved the ”Description of Rationale for Median Regression Analysis” from the ”Data Analysis Section” to the ”Results Section” and modified it as follows.

[***Pages 7-8, lines 244-279***: *To characterize research questions (1), (2) and (3), we perform regression analyses on the subjective happiness (SH), the revised generativity concern scale (GCS-R) and inquisitiveness are taken as dependent variables, respectively. When observations of these variables in the sample are considered to be non-normally distributed and/or skewed, the median regression is applied to analyze the determinants of dependent variables instead of parametric mean-based regressions, such as ordinary least squares (OLS) regression. We have run Shapiro-Wilk tests for three dependent variables of SH, GCS-R and inquisitiveness, as shown in Figure 2. The results reject the null hypothesis* ($z = 3.955, P < 0.01$) *for happiness,* ($z = 5.871, P < 0.01$) *for generativity and* ($z = 5.741, P < 0.01$) *for inquisitiveness. The tests confirm that the three variables, SH, GCS-R and inquisitiveness, are not normally distributed. The literature claims that median regressions are more appropriate than parametric mean-based ones, yielding robust estimations against the boundary values and/or outliers, especially when the dependent variable is bounded on a certain support range, non-normally distributed, and skewed [23, 63]. Therefore, we have employed the median regressions for happiness, generativity, and inquisitiveness with the specifications of Eq1 to 3, respectively.*] ■

(8) Thank you for pointing out my serious typo. We agree with you. Accordingly, We have corrected “generativity” to “subjective happiness (SH)” in the relevant sentence.

(9) Thank you for your comments. As you indicated, there was an error in the Figure number corresponding to paths. Accordingly, we have corrected the error so that the Figure numbers appear correctly.

(10) Thank you for your comments. We agree with you. Accordingly, we have corrected “Discussion” to ”Discussions” as you indicated.

(11) Thank you for your comments. We agree with your suggestion. Accordingly, we have replaced “open questions” with “research questions” in the relevant sentence.

(12) Thank you very much for your very kind suggestion. We agree with you. Accordingly, we have corrected the relevant text as follows. “Our study also demonstrates that children’s degree of inquisitiveness is significantly positively correlated with their generativity and happiness.”

[***Pages 18-19, lines 524-533***: *Hirose and Kotani [25] and Hirose et al. [26] have pointed out that inquisitiveness positively correlates with how people enhance their generative concern and happiness through questionnaire surveys with Japanese and Palauan adults, respectively. According to Hirose and Kotani [25] and Hirose et al. [26], inquisitiveness, directly and indirectly, affects happiness, but the most critical factor is generativity. Our study also demonstrates that children’s degree of inquisitiveness is positively correlated with their generativity and happiness. Some studies have pointed out that inquisitiveness is stable as a part*

of one's critical thinking disposition [5, 7, 3]. Conversely, other studies have pointed out that inquisitiveness can be acquired and further enhanced by learning [24, 18, 64, 19].] ■

(13) Thank you very much for your comments. Following your suggestion, we have modified the panel data to longitudinal data for the relevant parts.

(14) Thank you very much for your very kind advice. We have made a very elementary mistake. Accordingly, we have corrected the text in the diagram correctly.

References

1. Au, A., Lai, S., Wu, W., Hofer, J., Busch, H., Šolcová, I., Tavel, P., and Cheng, S.-T. (2020). Generativity and positive emotion in older adults: Mediation of achievement and altruism goal attainment across three cultures. *Journal of happiness studies*, 21:677–692.

2. Bibi, F., Chaudhry, A., and Awan, E. (2015). Impact of gender, age and culture on life satisfaction. *Science international*, 27:1649–1652.

3. Blickle, G., Meurs, J., Wihler, A., Ewen, C., and Peiseler, K. (2014). Leader inquisitiveness, political skill and follower attributions of leader charisma and effectiveness: Test of a moderated mediation model. *International journal of selection and assessment*, 22:272–285.

4. Chitchai, N., Senasu, K., and Sakworawich, A. (2018). The moderating effect of love of money on relationship between socioeconomic status and happiness. *Kasetsart journal of social sciences*, 2:1–9.

5. Chouinard, M., Harris, P., and Maratsos, M. (2007). Children's questions: A mechanism for cognitive development. *Monographs of the society for research in child development*, pages i–129.

6. Clark, S., Harbaugh, A., and Seider, S. (2019). Fostering adolescent curiosity through a question brainstorming intervention. *Journal of adolescence*, 75:98–112.

7. Cluver, A., Heyman, G., and Carver, L. (2013). Young children selectively seek help when solving problems. *Journal of experimental child psychology*, 115:570–578.

8. Diener, E. and Diener, M. (1995). Cross-cultural correlates of life satisfaction and self-esteem. *Journal of personality and social psychology*, 68:653–663.

9. Diener, E., Emmons, R., Larsen, R., and Griffin, S. (1985). The satisfaction with life scale. *Journal of personality assessment*, 49:71–75.

10. Diener, E., Oishi, S., and Lucas, R. (2003). Personality, culture, and subjective well-being: Emotional and cognitive evaluations of life. *Annual review of psychology*, 54:403–425.

11. Diener, E. and Ryan, K. (2009). Subjective well-being: A general overview. *South African journal of psychology*, 39:391–406.

12. Diener, E., Sapyta, J., and Suh, E. (1998). Subjective well-being is essential to well-being. *Psychological inquiry*, 9:33–37.

13. Diener, E., Suh, E., Lucas, R., and Smith, H. (1999). Subjective well-being: Three decades of progress. *Psychological bulletin*, 125:276–302.

14. Drenten, J., Peters, C., and Thomas, J. (2008). An exploratory investigation of the dramatic play of preschool children within a grocery store shopping context. *International Journal of retail & distribution management*, 36:831–855.

15. Engel, S. (2015). *The hungry mind: The origins of curiosity in childhood*. Harvard University Press.

16. Engelhard, G. and Monsaas, J. (1988). Grade level, gender and school-related curiosity in urban elementary schools. *The Journal of educational research*, 82:22–26.

17. Erikson, E. (1963). *Childhood and society*. Norton, second edition.

18. Frazier, B., Gelman, S., and Wellman, H. (2009). Preschoolers' search for explanatory information within adult-child conversation. *Child development*, 80:1592–1611.

19. Fusaro, M. and Smith, M. (2018). Preschoolers' inquisitiveness and science-relevant problem solving. *Early childhood research quarterly*, 42:119–127.

20. Gilbert, A., Colley, K., and Roberts, D. (2016). Are rural residents happier? A quantitative analysis of subjective wellbeing in Scotland. *Journal of rural studies*, 44:37–45.

21. Graesser, A. and Person, N. (1994). Question asking during tutoring. *American educational research journal*, 31:104–137.

22. Hagtvedt, L., Dossinger, K., Harrison, S., and Huang, L. (2019). Curiosity made the cat more creative: Specific curiosity as a driver of creativity. *Organizational behavior and humen decision processes*, 150:1–13.

23. Hao, L. and Naiman, D. (2007). *Quantile regression*, volume 149. Sage publications.

24. Hirayama, R. and Kusumi, T. (2004). Effect of critical thinking disposition on interpretation of controversial issues: Evaluating evidences and drawing conclusions. *Japanese journal of educational psychology*, 52:186–198.

25. Hirose, J. and Kotani, K. (2022). How does inquisitiveness matter for generativity and happiness? *PLoS ONE*, 17:e0264222.

26. Hirose, J., Kotani, K., and Managi, S. (2023). Do autonomy and inquisitiveness contribute to SDGs? Implications from the matrilineal island of Palau. *Economic Analysis and Policy*, 79:303–318.

27. Hofer, J., Busch, H., Chasiotis, A., Kärtner, J., and Campos, D. (2008). Concern for generativity and its relation to implicit pro-social power motivation, generative goals, and satisfaction with life: A cross-cultural investigation. *Journal of personality*, 76:1–30.

28. Huta, V. and Zuroff, D. (2007). Examining mediators of the link between generativity and well-being. *Journal of adult development*, 14:47–52.

29. Jan, M. and Masood, T. (2008). An assessment of life satisfaction among women. *Studies on home and community science*, 2:33–42.

30. Jorgensen, B., Jamieson, R., and Martin, J. (2010). Income, sense of community and subjective well-being: Combining economic and psychological variables. *Journal of economic psychology*, 31:612–623.

31. Kahneman, D., Diener, E., and Schwarz, N. (1999). *Well-being: Foundations of hedonic psychology*. Russell sage foundation.

32. Kashdan, T., Afram, A., Brown, K., Birnbeck, M., and Drvoshanov, M. (2011). Curiosity enhances the role of mindfulness in reducing defensive responses to existential threat. *Personality and individual differences*, 50:1227–1232.

33. Kashdan, T., Gallagher, M., Silvia, P., Winterstein, B., Breen, W., Terhar, D., and Steger, M. (2009). The curiosity and exploration inventory-II: Development, factor structure, and psychometrics. *Journal of research in personality*, 43:987–998.

34. Keller, M. and Ruus, R. (2014). Pre-schoolers, parents and supermarkets: co-shopping as a social practice. *International journal of consumer studies*, 38:119–126.

35. Lagattuta, K. and Wellman, H. (2002). Differences in early parent-child conversations about negative versus positive emotions: Implications for the development of psychological understanding. *Development psychology*, 38:564–580.

36. Lee, D. Y., Park, S. H., Uhlemann, M., and Patsult, P. (2000). What makes you happy? : A comparison of self-reported criteria of happiness between two cultures. *Social indicators research*, 50:351–362.

37. Lee, M.-A. and Kawachi, I. (2019). The keys to happiness: Associations between personal values regarding core life domains and happiness in South Korea. *PLoS ONE*, 14:e0209821.

38. Lepper, M., Corpus, J., and Iyengar, S. (2005). Intrinsic and extrinsic motivational orientations in the classroom: Age differences and academic correlates. *Journal of educational psychology*, 97:184–196.

39. Leung, A., Kier, C., Fung, T., Fung, L., and Sproule, R. (2011). Searching for happiness: The importance of social capital. *Journal of happiness studies*, 12:443–462.

40. Luhmann, M., Schimmack, U., and Eid, M. (2011). Stability and variability in the relationship between subjective well-being and income. *Journal of rural studies*, 45:186–197.

41. Lyubomirsky, S. and Lepper, H. (1999). A measure of subjective happiness: Preminary reliability and construct validation. *Social indicators research*, 46:137–155.

42. Magnani, E. and Zhu, R. (2018). Does kindness lead to happiness? Voluntary activities and subjective well-being. *Journal of behavioral and experimental economics*, 77:20–28.

43. Marshall, D. (2014). Co-operation in the supermarket aisle: Young children's accounts of family food shopping. *International journal of retail & distribution management*, 42:990–1003.

44. Maslow, A. (1954). *Motivation and personality*. Harper & Row publisher.

45. McAdams, D. and Aubin, E. (1992). A theory of generativity and its assessment through self-report, behavioral acts, and narrative themes in autobiography. *Journal of personality and social psychology*, 62:1003–1015.

46. Meisenberg, G. and Woodley, M. (2015). Gender differences in subjective well-being and their relationships with gender equality. *Journal of happiness studies*, 16:1539–1555.

47. Mentzakis, E. and Moro, M. (2009). The poor, the rich and the happy: Exploring the link between income and subjective well-being. *Journal of socio-economics*, 38:147–158.

48. Minahan, S. and Huddleston, P. (2013). Shopping with my mother: Reminiscences of adult daughters. *International journal of consumer studies*, 37:373–378.

49. Oishi, S. and Diener, E. (2009). Goals, culture, and subjective well-being. In *Culture and well-being*, pages 93–108. Springer.

50. Pelz, M. and Kidd, C. (2020). The elaboration of exploratory play. *Philosophical transactions of the royal society B*, 375:20190503.

51. Pluut, H. and Wonders, J. (2020). Not able to lead a healthy life when you need it the most: Dual role of lifestyle behaviors in the association of blurred work-life boundaries with well-being. *Frontiers in psychology*, 11:607294.

52. Salavera, C., Usán, P., and Teruel, P. (2020). The mediating role of positive and negative affects in the relationship between self-esteem and happiness. *Psychology research and behavior management*, 13:355–361.

53. Schoklitsch, A. and Baumann, U. (2012). Generativity and aging: A promising future research topic? *Journal of aging studies*, 26:262–272.

54. Shahen, M., Shahrier, S., and Kotani, K. (2019). Happiness, generativity and social preferences in a developing country: A possibility of future design. *Sustainability*, 11:5256.

55. Silvia, P. and Kashdan, T. (2009). Interesting things and curious people: Exploration and engagement as transient states and enduring strengths. *Social and personality psychology compass*, 3:785–797.

56. Timilsina, R., Kotani, K., and Kamijo, Y. (2019). Generativity and social value orientation between rural and urban societies in a developing country. *Futures*, 105:124–132.

57. Tizard, B. and Hughes, M. (2008). *Young children learning*. John Wiley & Sons.

58. Tkach, C. and Lyubomirsky, S. (2006). How do people pursue happiness?: Relating personality, happiness-increasing strategies and well-being. *Journal of happiness studies*, 7:183–225.

59. Veenhoven, R. (1991). Is happiness relative? *Social indicators research*, 24:1–34.

60. Villar, F. (2012). Successful ageing and development: The contribution of generativity in older age. *Aging & society*, 32:1087–1105.

61. Warner, R. and Vroman, K. (2011). Happiness inducing behaviors in everyday life: An empirical assessment of "the how of happiness". *Journal of happiness studies*, 12:1063–1082.

62. Welsch, H. (2006). Environment and happiness: Valuation of air pollution using life satisfaction data. *Ecological economics*, 58:801–813.

63. Wooldridge, J. (2019). *Introductory econometrics: A modern approach*. Cengage Learning, seventh edition.

64. Yamaguchi, Y. and Sannomiya, M. (2012). Beliefs and attitudes about creativity among Japanese university students. *Creativity & human development*, 14:1–11.

65. Zidansek, A. (2007). Sustainable development and happiness in nations. *Energy*, 32:891–897.

List of Figures

1 A conceptual framework describing the relationships concerning subjective happiness (SH) among cognitive, noncognitive and sociodemographic factors 17
2 Histgrams of the dependent variables 18

Figure 1: A conceptual framework describing the relationships concerning subjective happiness (SH) among cognitive, noncognitive and sociodemographic factors

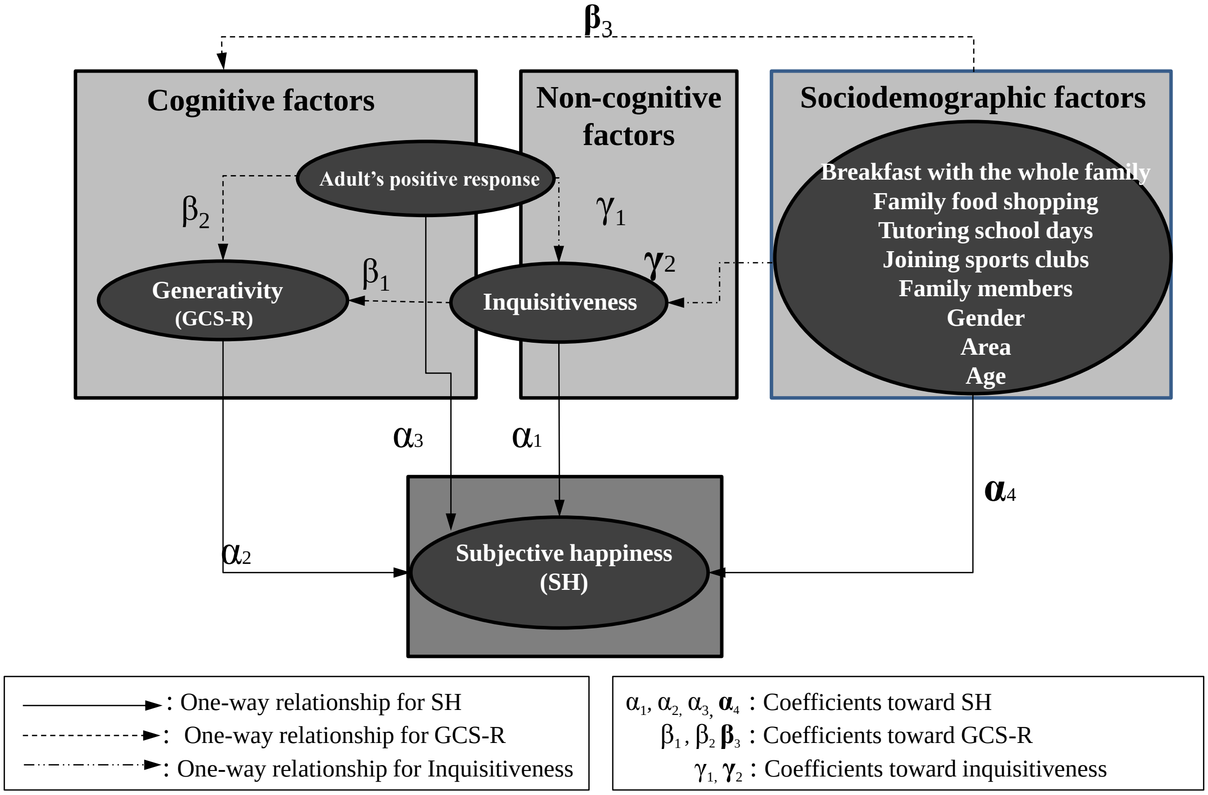

Fig. 1: A conceptual framework describing the relationships concerning subjective happiness (SH) among cognitive, noncognitive and sociodemographic factors.

Figure 2: Histgrams of the dependent variables

(a) Happiness (SH)

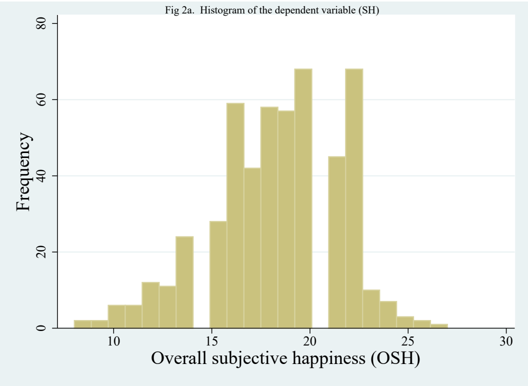

(b) Generativity (GCS-R)

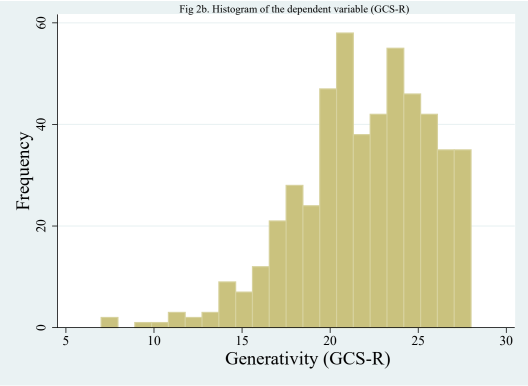

(c) Inquisitiveness

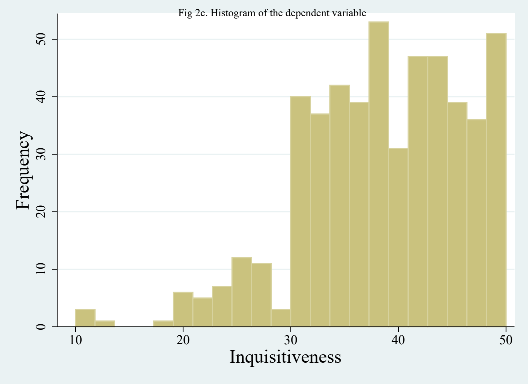

Supplement: Attachment — Submitted filename: Response _To_Reviewer_2.pdf [file pone.0303523.s003.pdf]
